# Supplementary material for: Sorting at embryonic boundaries requires high heterotypic interfacial tension
Source: Nat Commun. 2017 Jul 31;8:157. doi: 10.1038/s41467-017-00146-x (PMC5537356; doi:10.1038/s41467-017-00146-x)
Supplement: Supplementary file 2 — Supplementary Software 1 [file 41467_2017_146_MOESM2_ESM.zip › PottsModel/SrcPottsModel/doc/model/EnergyFunction.html]

EnergyFunction


JavaScript is disabled on your browser.


Skip navigation links


- Overview
- Package
- Class
- Use
- Tree
- Deprecated
- Index
- Help

- Prev Class
- Next Class

- Frames
- No Frames

- All Classes

- Summary:
- Nested |
- Field |
- Constr |
- Method

- Detail:
- Field |
- Constr |
- Method


model

## Interface EnergyFunction

- All Known Implementing Classes:
  :   AreaEnergyFunction, InterfacialTensionEnergyFunction, PerimeterEnergyFunction, PixelInteractionEnergyFunction

  ---

    

  ```
  public interface EnergyFunction
  ```

- - ### Nested Class Summary

    Nested Classes

    | Modifier and Type | Interface and Description |
    | --- | --- |
    | `static class` | `EnergyFunction.Energy` |
  - ### Method Summary

    All Methods Instance Methods Abstract Methods

    | Modifier and Type | Method and Description |
    | --- | --- |
    | `double` | `getDeltaEnergy(Pixel s, Pixel t)` |
    | `EnergyFunction.Energy` | `getEnergyType()` |
    | `java.lang.String` | `getName()` |

- - ### Method Detail


    - #### getEnergyType

      ```
      EnergyFunction.Energy getEnergyType()
      ```


    - #### getName

      ```
      java.lang.String getName()
      ```


    - #### getDeltaEnergy

      ```
      double getDeltaEnergy(Pixel s,
                            Pixel t)
      ```


Skip navigation links


- Overview
- Package
- Class
- Use
- Tree
- Deprecated
- Index
- Help

- Prev Class
- Next Class

- Frames
- No Frames

- All Classes

- Summary:
- Nested |
- Field |
- Constr |
- Method

- Detail:
- Field |
- Constr |
- Method
